# Inorganic Phosphate Accelerates the Migration of Vascular Smooth Muscle Cells: Evidence for the Involvement of miR-223

**DOI:** 10.1371/journal.pone.0047807

**Published:** 2012-10-18

**Authors:** Ashraf Yusuf Rangrez, Eléonore M'Baya-Moutoula, Valérie Metzinger-Le Meuth, Lucie Hénaut, Mohamed Seif el Islam Djelouat, Joyce Benchitrit, Ziad A. Massy, Laurent Metzinger

**Affiliations:** 1 INSERM U1088, Amiens, France; 2 Faculty of Pharmacy and Medicine, Jules Verne University of Picardie, Amiens, France; 3 Division(s) of Pharmacology / Nephrology, Amiens University Hospital, Amiens, France; 4 UFR SMBH, University of Paris 13, Bobigny, France; IRCCS-Policlinico San Donato, Italy

## Abstract

**Backgound:**

An elevated serum inorganic phosphate (Pi) level is a major risk factor for kidney disease and downstream vascular complications. We focused on the effect of Pi levels on human aortic vascular smooth muscle cells (VSMCs), with an emphasis on the role of microRNAs (miRNAs).

**Methodology/Principal Findings:**

Exposure of human primary VSMCs *in vitro* to pathological levels of Pi increased calcification, migration rate and concomitantly reduced cell proliferation and the amount of the actin cytoskeleton. These changes were evidenced by significant downregulation of miRNA-143 (miR-143) and miR-145 and concomitant upregulation of their targets and key markers in synthetic VSMCs, such as Krüppel-like factors−4 and −5 and versican. Interestingly, we also found that miR-223 (a marker of muscle damage and a key factor in osteoclast differentiation) is expressed in VSMCs and is significantly upregulated in Pi-treated cells. Over-expressing miR-223 in VSMCs increased proliferation and markedly enhanced VSMC migration. Additionally, we found that the expression of two of the known miR-223 targets, Mef2c and RhoB, was highly reduced in Pi treated as well as miR-223 over-expressing VSMCs. To complement these *in vitro* findings, we also observed significant downregulation of miR-143 and miR-145 and upregulation of miR-223 in aorta samples collected from ApoE knock-out mice, which display vascular calcification.

**Conclusions/Significance:**

Our results suggest that (i) high levels of Pi increase VSMC migration and calcification, (ii) altered expression levels of miR-223 could play a part in this process and (iii) miR-223 is a potential new biomarker of VSMC damage.

## Introduction

Vascular smooth muscle cells (VSMCs) are the predominant cells in the tunica media of arteries. They play a critical role in regulating the blood vessel tone, which in turn influences blood pressure. Vascular smooth muscle cells are not terminally differentiated and are able to switch from a differentiated (contractile) state to a dedifferentiated (synthetic) state in response to changing environmental signals. This phenotype modulation is critical in the pathogenesis of proliferative cardiovascular diseases (reviewed by House et al.[1]). The synthetic phenotype is concomitant with accelerated migration and ultimately results in the formation of vascular calcification - one of the main pathological lesions in chronic kidney disease[2]. Hyperphosphatemia is a major cause of this calcifying process[3], [4] and the latter is correlated with increased cardiovascular mortality in dialysis patients[2]. In the present work, we sought to determine whether or not microRNAs (miRNAs) play a role in the VSMC calcification process induced by inorganic phosphate (Pi).

MicroRNAs (miRNAs) are a novel class of small RNAs that negatively regulate gene expression *via* repression of the corresponding target mRNAs[5], [6]. Several miRNAs have been found to have roles in the healthy and diseased vascular system[6], [7]. MiR-143 and miR-145 (the most extensively studied species) have been correlated with human cardiovascular diseases, since VSMC maintenance and vascular homeostasis are altered in *mir-143* and *mir-145* knock-out (KO) mice[8]. The latter authors also determined that expression levels of miR-143 and miR-145 are low in the aortas of apolipoprotein E gene knockout (ApoE-KO) mouse. Boettger and colleagues also demonstrated that the mouse *mir-143/145* cluster is necessary for acquisition of the contractile VSMC phenotype[9]. Additionally, a detailed study by Cordes et al.[10] showed that miR-145 can direct the fate of smooth muscle and regulate the synthetic phenotype of smooth muscle cells.

In the present study, we investigated the molecular mechanisms by which Pi impacts on the phenotype of primary VSMC cultures derived from human aortas and correlated these effects with the expression of miRNAs. Evidence from our studies suggests that Pi alters cell proliferation and migration, reduces the amount of the actin cytoskeleton, downregulates miR-143 and miR-145 and upregulates miR-223. Indeed, increased levels of miR-223 have been reported in damaged skeletal muscle (in Duchenne muscular dystrophy[11]) and in cardiomyocytes[12]). Hence, we hypothesized that this miRNA might also be a marker of smooth muscle damage. Furthermore, miR-223 has been linked to osteogenesis and calcium-phosphate deposits(Ca*Pi) [13]. We found that upregulating miR-223 in VSMCs induces a significant increase in cell proliferation and migration and reduces the amount of the actin cytoskeleton. We also show that modulating the expression of miR-223 affects the expression of its reported targets Mef2c and RhoB in our *in vitro* model. Lastly, we detected the upregulation of miR-223 expression *in vivo*, in aorta collected from ApoE-KO mice (a well-established model of atherosclerosis and vascular calcification, displaying Ca*Pi [3]).

## Results

### Elevated Pi induces VSMC calcification and migration and reduces proliferation

**Figure 1:**
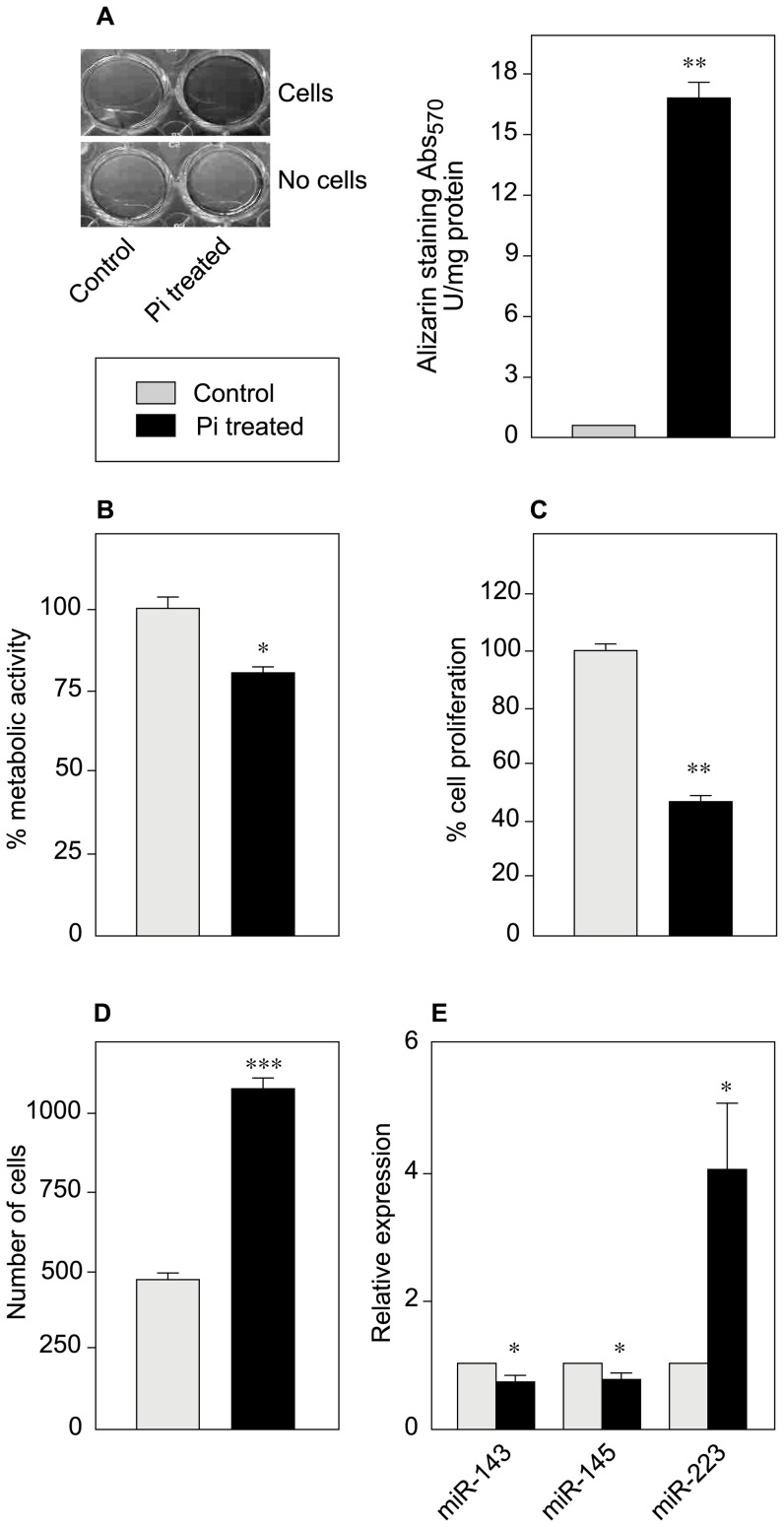
Physiological and molecular effects of Pi. VSMCs were cultured for 10 days in DMEM supplemented with 1% FBS in the presence or absence of 3.5 mM Pi. (**A**) A representative photograph of a calcification assay using alizarin staining, indicating greater calcification in Pi treated cells and the corresponding quantification. Alongside a control with cells incubated at 1.1 mM Pi and to check for non-specific calcium deposition, we also ran a cell-free control with the same Pi concentrations. (**B**) The VSMCs' metabolic activity was studied in a WST-1 colorimetric assay. Cells treated with 3.5 mM Pi showed low metabolic activity. (**C**) VSMC proliferation was measured in a BrdU ELISA. (**D**) The migration rate of VSMCs was determined using Boyden chambers and was found to be twice as high in Pi-treated VSMCs than in control cells. (**E**) Expression of miR-143, miR-145 and miR-223 was measured by qPCR. Data are represented as the mean of two or three independent experiments in triplicate.

Hyperphosphatemia is associated with vascular calcification in various cardiovascular disorders[14]. In order to study the direct effect of Pi on VSMCs from the human aorta, we treated the cells for 10 days with 3.5 mM Pi (to reflect hyperphosphatemia [15]) in Dulbecco's modified Eagle Medium (DMEM) with 1% Fetal Bovine Serum (FBS; the concentration needed to obtain Ca*Pi deposits *in vitro*). We determined the level of calcification by alizarin staining and found that Pi indeed induced calcification in VSMCs. The calcification was 15- to 20-fold higher in Pi treated-cells than in control cells exposed to a physiological concentration of Pi (1.1 mM) (Figure 1A). The same Pi concentration significantly lowered the VSMCs' metabolic activity (by 25%, as measured in a WST-1 assay; Figure 1B) and proliferation activity (by 50%, as measured by BrdU incorporation; Figure 1C). Decreased proliferation induced by high Pi treatment could tentatively be attributed to increased apoptosis. In order to explore this hypothesis, we carried out Annexin V/PI staining. Our results indicate that Pi treatment for 10 days does not affect apoptosis rate in our experimental conditions (figure S1). Other, as yet undetected mechanisms are thus responsible for this phenomenon.

We next looked at whether high Pi had an effect on VSMC migration. We treated VSMCs for 3 days with Pi at 3.5 mM and then studied their migration rate using Boyden chambers. These experiments were performed in the presence of 10% FBS - the concentration needed to obtain significant cell migration. Interestingly, our results indicated that a high Pi level significantly increased the rate of VSMC migration (approximately 2.5-fold, relative to controls; Figure 1D).

### Elevated Pi significantly downregulates expression of miR-143 and miR-145 and upregulates miR-223

Many recent studies have investigated and confirmed the role of miR-143 and miR-145 in regulating the balance between the VSMC's contractile phenotype and vascular calcification[7], [8], [9], [10]. Low levels of these miRNAs are also associated with coronary artery disease[16]. We thus investigated the expression of miRNAs miR-143 and miR-145 in the presence of high Pi. There was a significant downregulation (20–25%) of both miR-143 and miR-145 in 3.5 mM Pi treated cells, when compared with control cells (Figure 1E).

We also determined the effect of Pi on expression levels of the osteogenesis marker miR-223[13], which is also a newly described marker of muscle damage[11], [12]. To the best of our knowledge, this is the first report of expression of miR-223 in VSMCs (Figure 1E and Figure S2). Moreover, quantitative real-time PCR evidenced strong (4-fold) upregulation of miR-223 in Pi-treated cells (Figure 1E); this finding was confirmed by *in situ* Hybridization (ISH) (Figure S2).

Additionally, we normalized the expression of miR-223 to the expression of the VSMC-specific miR-143 (Figure S3). The relative expression of miR-223 compared to miR-143 at basal level, i.e. in control cells is approximately 1000 times lower than the VSMC specific miR-143. However, impressively, in Pi treated cells, the expression of miR-223 increased dramatically and the relative expression increased up to 700% fold. This is suggestive of the role of miR-223 in VSMC calcification.

### Elevated Pi modulates the expression of phenotypic marker genes and the amount of actin cytoskeleton

**Figure 2:**
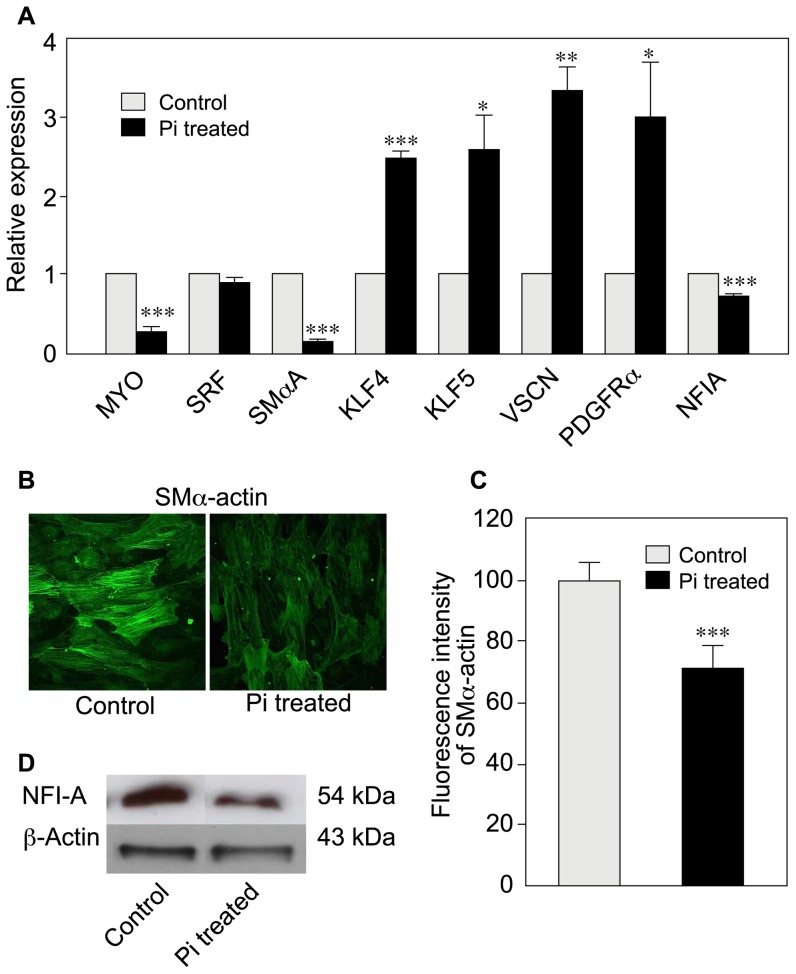
Elevated Pi affects VSMC phenotypic markers. VSMCs were cultured for 10 days in DMEM supplemented with 1% FBS, in the presence or absence of 3.5 mM Pi. (A) After RNA extraction, we performed RT-qPCR using specific primers for VSMC phenotypic marker genes. Contractile phenotype markers (such as myocardin (MYO) and Smooth muscle α–actin (SMαA) were highly downregulated), whereas synthetic phenotype markers like Krüppel-like factor 4 (KLF4), KLF5, versican (VSCN) and platelet-derived growth factor receptor-α (PDGFRα) were significantly upregulated (n = 3). (B) Immunofluorescence of microscopic images showing the actin cytoskeleton after Pi treatment of VSMCs (magnification 40X), as also assessed (C) by fluorescence intensity measurements in corresponding histogram. (D) Representative immunoblot indicating the downregulation of NFIA in Pi-treated VSMCs. β-actin was used as endogenous control. One representative experiment shown out of two or three independent experiments.

Given our observation of significant downregulation of miR-143 and miR-145 and upregulation of miR-223 in cells treated with 3.5 mM Pi, we used RT-qPCR to determine the expression levels of representative VSMC phenotypic marker genes - most of which are targeted by these miRNAs[8], [10]. We found that Pi treatment results in significant downregulation of contractile marker genes (Figure 2A), such as myocardin (MYO) and Smooth Muscle α–actin (SMαA). Nuclear factor IA (NFIA, an experimentally validated miR-223 target[13]) appeared to be downregulated in cells treated with 3.5 mM Pi, as expected (given the increase in miR-223). The low level of NFIA was confirmed by Western blotting (Figure 2D). In contrast, some of the synthetic phenotypic markers (such as Krüppel-like factor 4 [KLF4], KLF5, platelet derived growth factor receptor-α [PDGFR α] and versican [VSCN]) which are all targeted by miR-143 and/or miR-145[7] were significantly upregulated; this was expected, given the observed downregulation of the corresponding miRNAs (Figure 2A).

In view of the significant downregulation of the mRNA coding for SMαA in the qPCR, we performed immunocytochemistry using a SMαA-specific antibody to assess the impact of elevated Pi on the actin cytoskeleton. As shown in Figures 2B and C and Figure S4, treatment with 3.5 mM Pi drastically affected the organization of the actin cytoskeleton and, in turn, the morphology of the VSMCs.

Furthermore, we also performed an immunostaining against CTTN, one of the podosome markers. As adhesion structures, podosomes play an important role in cell migration, tissue invasion, extracellular matrix degradation. They are the primary sites of integrin-stimulated actin polymerization, and allegedly contribute to cellular invasiveness in physiological and pathological situations [17]. In Figure S5, we observed that 3.5 mM Pi-treated VSMCs exhibit an increased number of podosomes when compared with cells treated with a physiological, 1.1 mM Pi concentration. Also, Figure S5 clearly shows that the CTTN stained, podosome rich VSMCs are deprived of SMαA further validating the qPCR and SMαA staining results.

### miR-223 increases VSMC migration and proliferation and decreases the amount of actin cytoskeleton

**Figure 3:**
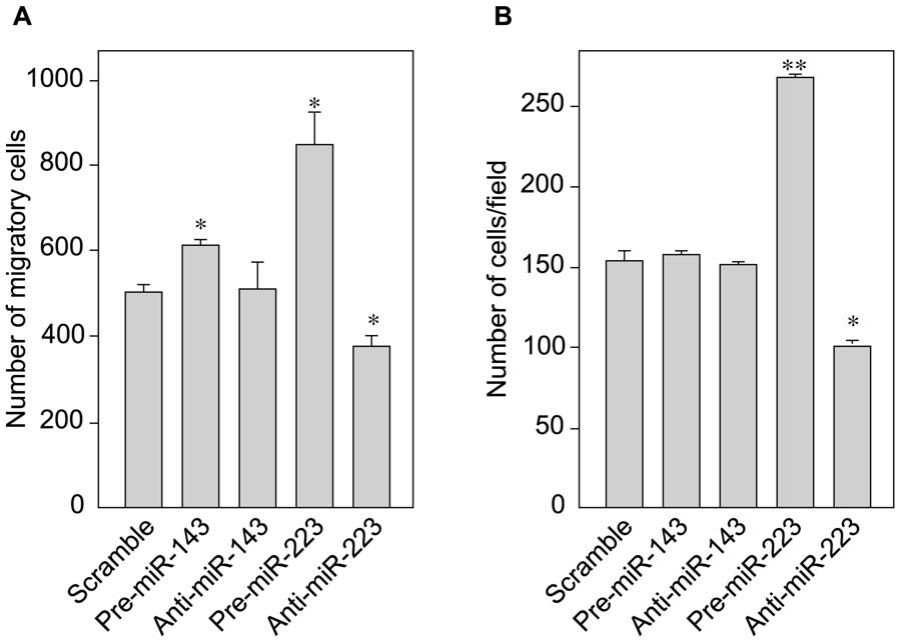
Over-expression of miR-223 enhances VSMC migration. To upregulate and knock-down the expression of miR-223, VSMCs were transfected for 48 h with pre-miR-223 and anti-miR-223, respectively. Scrambled, unrelated miRNA was used as a control. The migration rate was studied by (A) wound healing assay and (B) Boyden chambers (n = 3, two independent experiments).

To further study the effects of miR-143 or miR-223, we used a wound-healing assay that mimics cell motility during vessel healing *in vivo* and enables migration to be studied. We created a “wound” in the monolayer and captured the images of VSMC migration after 48 h in culture. Vascular smooth muscle cells transfected with pre-miR (over-expression) and anti-miR (knock-down) specific for miR-143 and miR-223 were compared with a scrambled RNA control (Figure 3A). Anti-miR-143 treatment did not affect motility, whereas pre-miR-143 treatment was associated with moderately (20%) but significantly greater migration than in control experiments. Pre-miR-223 induced a highly significant 64% increase in cell migration. Accordingly, anti-miR-223 treatment was associated with significantly (28%) lower migration (Figure 3A).

In Figure S6, we show that, as previously described in Figure 3, mir-223 overexpression increases VSMC migration, mir-223 inhibition decreases VSMC migration, and high Pi treatment (3.5 mM) increases VSMC migration. Interestingly, high Pi attenuates the effect of miR-223 inhibition on migration, and the levels of the combination of both treatments are comparable to control levels, *ie* high Pi neutralizes the effect of anti-miR-223. Additionally, neither anti-miR-143, anti-miR-145, pre-miR-143 nor pre-miR-145 treatment induced any change in this migration assay (Figure S6). We also measured the expression level of the marker SMαA in the same conditions: as previously show, high concentrations of Pi reduced the expression of SMαA. However, combining Pi and anti-miR-223 had the same effect as anti-miR-223 alone compared to control. This indicates that anti-miR-223 and high Pi neutralize each other's effect on both VSMC migration and SMαA expression (data not shown).

Boyden chambers enable directional cell migration to be quantified (Figure 3B). This independent and complementary technique yielded similar results after 5 h of migration: pre-miR-223 was associated with significantly greater (75%) cell migration, vs. controls. Furthermore, anti-miR-223 treatment was associated with a VSMC migration rate that was 33% lower than for controls (Figure 3B). Neither anti-miR-143 nor pre-miR-143 treatment induced any change in the results for this assay.

**Figure 4:**
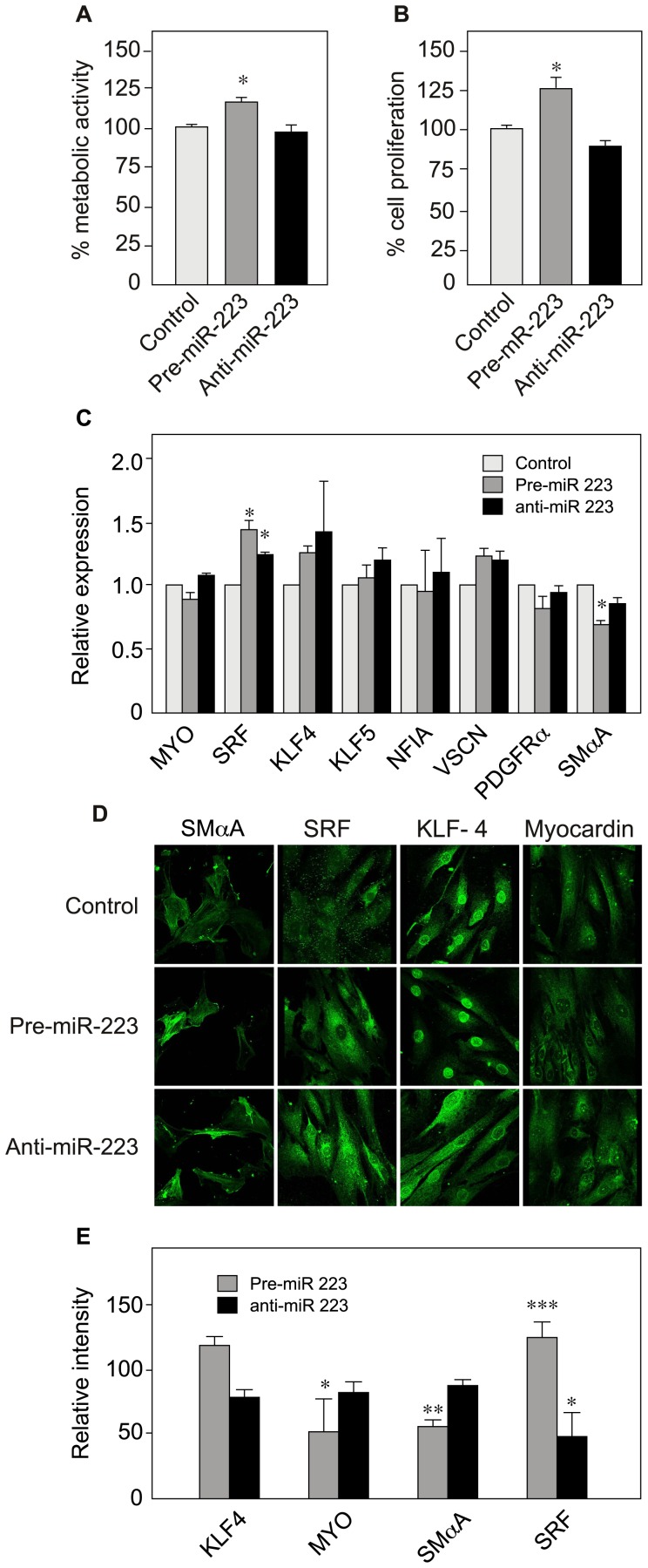
Effect of miR-223 up- and downregulation on VSMC proliferation, cytoskeleton size and marker gene expression. To upregulate and knock-down the expression of miR-223, VSMCs were transfected using pre-miR-223 and anti-miR-223, respectively. After 48h of transfection, cells were assayed for metabolic activity (A), cell proliferation (B) and marker gene expression levels (with RT-qPCR) (C). Data are presented as the mean ± SD of triplicates. One representative experiment is shown (n = 2). (D) Immunostaining of VSMC protein markers. (E) Quantification of the immunofluorescence data from Fig. 4D. Data are presented as the mean ± SD relative to the control (scrambled RNA, set at 100%) (KLF4 n = 12, MYO n = 11, SMαA n = 5, SRF n = 8). See Fig. 2 for abbreviations.

We thus evidenced a significant impact of miR-223 on VSMC migration. We decided to focus on this miRNA by modulating its expression and studying the effects on cell proliferation and marker gene expression. Relative to control experiments, over-expression of miR-223 significantly increased the metabolic activity of VSMCs (by 17%, according to the WST-1 assay). However, miR-223 knock-down had no effect (Figure 4A). We found similar results (a 25% increase) for miR-223 upregulation in the BrdU assay, which measures DNA synthesis and thus VSMC proliferation. Again, knock-down had no effect (Figure 4B). We next looked at the effect of miR-223 regulation on several VSMC marker genes (Figure 4C). Interestingly, miR-223 upregulation induced a significant downregulation of SMαA mRNA and a mild increase in Serum Response Factor (SRF) mRNA but did not significantly affect other marker genes. This was further confirmed by immunofluorescence protein staining, which clearly showed a very low level of SMαA and MYO and a high level of SRF when miR-223 was over-expressed in VSMCs (Figure 4D-E). In contrast, anti-miR treatment was associated only with significantly lower levels of SRF (Figure 4D -E).

### miR-223 upregulation modulates the expression of target proteins mef2c and Rhob

**Figure 5:**
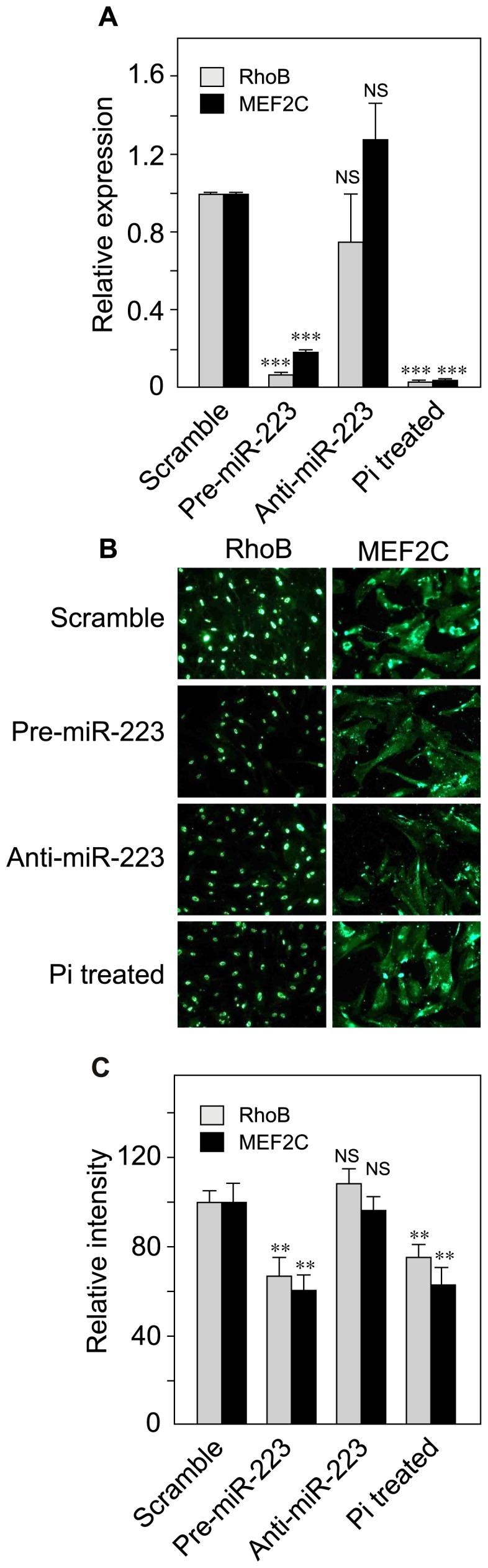
Effect of miR-223 up- and down regulation and Pi on miR-223 specific targets Mef2c and RhoB. To up-regulate and knock-down the expression of miR-223, VSMCs were transfected for 48 h with pre-miR223 and anti-miR-223, respectively. Scrambled, unrelated miRNA was used as a control. For Pi effect, VSMCs were cultured for 2 days in DMEM supplemented with 1% FBS, in the presence or absence of 3.5 mM Pi. (A) After RNA extraction, expression of Mef2c and RhoB messengers was measured by RT-qPCR. Data are represented as the mean of 3 independent experiments +/− SD). (B) Immunofluorescence images showing the effect of miR-223 up- and down regulation and Pi on Mef2c and RhoB protein expression in VSMCs (magnification 20X). (C) Fluorescence intensity measurements from the immunofluorescence images displayed in Figure 4B, showing the effect of miR-223 up- and down regulation and Pi on Mef2c and RhoB protein expression (n = 3 for each marker, data are represented as the mean of 3 independent experiments +/− SD).

We next investigated the effect of miR-223 regulation on reported targets Mef2c and RhoB. Mef2c has been shown to play a critical role in VSMC differentiation and to regulate myocardin expression [18].Two independent studies have validated that miR-223 directly targets and inhibits Mef2c mRNA [19]–[20]. Accordingly, in our model, miR-223 upregulation induced a strong and significant decrease of Mef2c mRNA (of approximately 80%, Figure 5A) and a concomitant decrease of the protein product, as evidenced by immunofluorescence protein staining (of approximately 35%, Figure 5B and C). Interestingly, high Pi treatment induced comparable changes, suggesting a link between the two treatments. On the other hand, miR-223 knock-down had no effect on the expression of these markers. We also looked at the effect of miR-223 regulation on another recently described miR-223 target, RhoB [21]. RhoB is a member of the Rho guanosine triphosphatases family of proteins which has been shown to increase VSMC contractility and mediate adaptational changes to hypoxia [22]. Like with Mef2c, miR-223 upregulation significantly decreased the amount of mRNA (of approximately 90%, Figure 5A) with a concomitant decrease of the RhoB protein (of approximately 40%, Figure 5B and C). Again, 3.5 mM Pi treatment also induced very similar dramatic changes in RhoB gene expression (90% decrease of mRNA amount, and 40% decrease of the protein). As in the Mef2c experiments, anti-miR-223 treatment had no detectable effect.

### miR-143 and miR-145 are downregulated and miR-223 is upregulated in ApoE-KO mice

**Figure 6:**
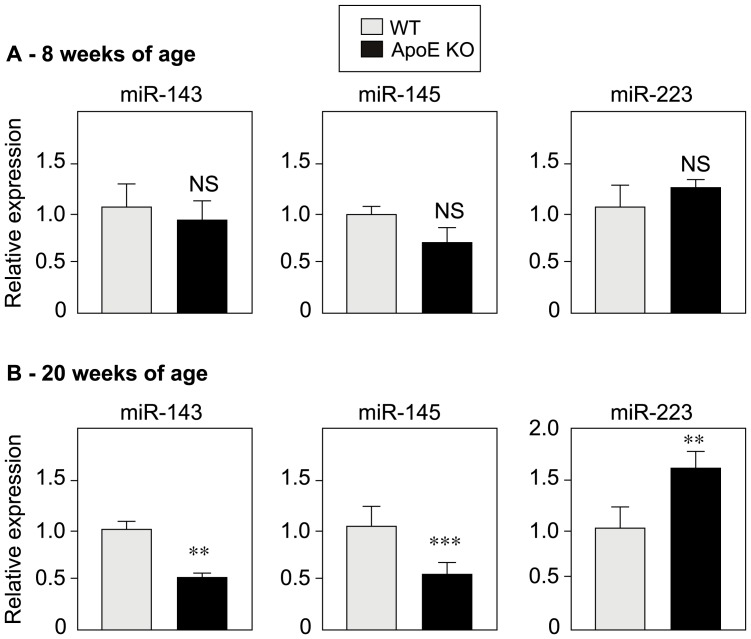
Expression of miR-143, miR-145 and miR-223 in wild-type and ApoE-KO mice. RNAs extracted from mouse aorta collected from 8- and 20-week-old ApoE-KO and wild-type mice were used for the qPCR expression analysis of miR-143, miR-145 and miR-223. There was significant downregulation of miR-143 and miR-145 and concomitant upregulation of miR-223 in 20-week-old ApoE-KO mice. Data are presented as the mean ± SD of 4 mice for each group.

Lastly, we sought to study the fate of miR-143, miR-145 and miR-223 under *in vivo* vascular calcification conditions. We evaluated the levels of these miRNAs in aorta isolated from 8-week-old and 20-week-old ApoE-KO and WT mice from the same genetic background (C57BL6J, Figure 6). Our group has earlier published that 20-week-old ApoE-KO mice display more vascular calcification than WT mice[23]. The disease state in ApoE-KO mice was checked by determining blood cholesterol and triglycerides, which were constantly elevated (not shown). No significant differences in expression of either of the miRNAs were observed in young, 8-week-old animals (Figure 5A). In contrast, significantly greater expression of miR-223 (1.6-fold) was observed in older, 20-week-old ApoE-KO mice (which displayed vascular calcification), relative to WT mice (Figure 5B). As observed with VSMCs in culture, the expression levels of both miR-143 and miR-145 were significantly lower in ApoE-KO mice than in WT mice. This highly significant, approximately 2-fold difference was observed at the age of 20 weeks (Figure 5B). Our *in vivo* results in a well-established murine model thus reflected our *in vitro* findings, i.e. downregulation of miR-143 and miR-145 and upregulation of miR-223 in the presence of calcium-Pi deposits.

## Discussion

Contractile VSMCs are crucial for maintaining arterial wall structural integrity and function. However, in certain vascular diseases (such as atherosclerosis), impaired contractility leads to calcification and thickening. Many recent studies have shown that an elevated serum Pi level is one of the causative agents of (and is a risk factor for) vascular complications[2], [3]. We thus examined the direct effect of Pi on the pathophysiology of human aortic VSMCs on the cellular and molecular levels. We observed an induction of calcification and an increased migration rate in VSMCs exposed to a pathological concentration of Pi, with a concomitant reduction in cell proliferation and an alternation in the organisation of the actin cytoskeleton. The lower proliferation rate can be correlated with increased cytotoxicity and a greater rate of apoptosis, which Pi reportedly induces in VSMCs[24]. We found significant downregulation of the vascular miRNAs miR-143 and miR-145 and a number of contractile phenotypic marker genes, such as MYO and SMαA[7]–[10]. A key finding of our study was that miR-223 (which is conventionally associated with carcinogenesis[25]–[26]but is also a newly described marker of skeletal and cardiac muscle damage [11]–[12] is expressed in VSMCs and is strongly upregulated in calcifying VSMCs and in calcified aortas from ApoE-KO mice. Finally, we show strong reduction in the expression of two of miR-223 targets, Mef2c and RhoB, which have established roles in VSMC biology.

**Figure 7:**
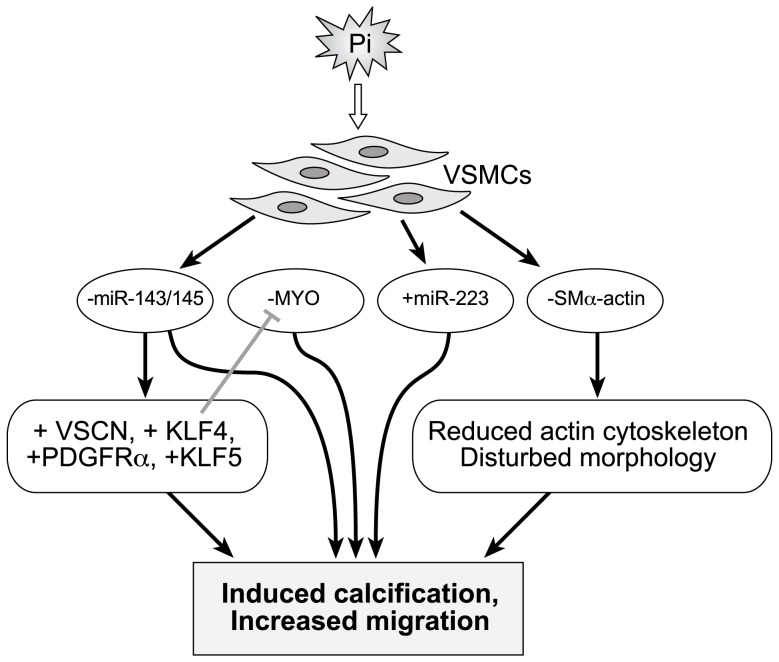
Schematic representation of the hypothetical molecular and cellular consequences of elevated phosphate exposure in VSMCs. Elevated extracellular Pi resulted in the downregulation of VSMC master regulators, MYO and SMαA. The expression of miR-143 and miR-145 was also downregulated in Pi-treated cells. These events affected downstream processes by reducing the size of the actin cytoskeleton, disturbing cell morphology, upregulating miR-143 and miR-145 targets and, ultimately, leading to increased calcification and a greater VSMC migration rate. Up-regulation of miR-223 further enhances VSMC migration and reduces the amount of actin cytoskeleton. ‘-’ indicates downregulation, whereas ‘+’ indicates upregulation of the respective miRNAs or genes. See Fig. 2 for abbreviations.

Another group of researchers has already demonstrated that high Pi concentrations are associated with greater calcification in VSMCs[27]. Our data confirm these literature results and further demonstrate that hyperphosphatemia increases the VSMCs' ability to migrate and reduces cell metabolic activity and proliferation. Based on earlier reports, we postulated that the reduced proliferation in Pi treated VSMCs could be explained by increased cytotoxicity and rate of apoptosis. However, we did not observe significant difference in the apoptosis rate in Pi treated or untreated cell in our experimental conditions. Therefore, the increased calcification and some yet unknown mechanism would be involved in lowering the cell proliferation in Pi treated VSMCs. Interestingly though, our results indicate that hyperphosphotemia at least contributes to inducing VSMCs to switch from a differentiated state to a dedifferentiated state by modulating the underlying molecular markers (Figure 7).

MiR-143 and miR-145 negatively regulate the expression of many genes that are specific for the VSMC synthetic phenotype[7], [10]. Here, we showed that high Pi treatment results into downregulation of SMαA and MYO, with a concomitant downregulation of miR-143 and miR-145. Like MYO, SMαA is a VSMC differentiation marker and changes in its expression strongly affect the muscle cells' cytoskeleton and morphology[28]. Furthermore, downregulation of miR-143 and miR-145 in the presence of high Pi implies the upregulation of their targets (such as KLF4, KLF5, PDGFRα and VSCN). KLF4 is generally upregulated following vascular injury. It interacts with SRF and directly represses MYO gene expression[29]. KLF5 is an essential cardiovascular transcription factor. Its expression is strongly induced in activated VSMCs in atherosclerosis [30]. The significant upregulation of both KLF4 and KLF5 found in this study correlates well with the increased calcification and MYO downregulation. Recently, the chondroitin sulfate proteoglycan VSCN was identified as a new target for miR-143 [31]. It is produced by synthetic VSMCs and promotes VSMC migration and proliferation. Similarly, PDGFRα is a direct target of miR-143 and is one of the factors required for VSMC migration [32]. Hence, a greater migration rate could be due (at least in part) to upregulation of these factors. Interestingly, we also found that miR-143 and miR-145 are downregulated *in vivo* in the aortas of ApoE-KO mice. We thus confirmed the results of Elia et al.[8], who have reported a decrease in these miRNAs in vascular diseases (including in ApoE-KO mice). Here, we expanded on Elia et al. results by finding that downregulation of miR-143 and miR-145 in mouse aortas was not detected in younger mice but became significant in 20-week-old mice (which also display vascular calcification[23]).

One of our study's most important findings was the upregulation of miR-223 in VSMCs treated with high levels of Pi and in ApoE-KO mice displaying vascular calcification. miR-223 was first described as a myeloid-specific miRNA[12] and as a biological marker for several tumoral processes[25]–[26]. It has also been described as a marker of damage to both skeletal[11] and cardiac muscle tissues[12]. Here, we found that miR-223 was expressed in smooth muscle and that its upregulation is a potential marker of VSMC damage. We attempted to determine miR-223′s role in VSMC biology and showed that overexpressing this species increased VSMC proliferation. – an observation that fits well with miR-223′s known involvement in many tumoral processes [25]–[26]. In the present study, we used two independent techniques to show that miR-223 over-expression had a significant impact on VSMC migration. Again, this agrees with studies correlating miR-223 over-expression with increased tumour cell migration[20], [26]. To the best of our knowledge, ours is the first report of an effect of miR-223 on migration in normal (i.e. non-tumor) cells. Furthermore, we detected downregulation of SMαA at both the mRNA and protein levels during overexpression of miR-223. This alteration in the cytoskeleton may be related to the observed, Pi-induced changes in the proliferative and migrative activities of VSMCs. Similarly, we saw significant downregulation of MYO (which is involved in cell migration and proliferation[8]–[10] when miR-223 was over-expressed. This was concomitant with an increase in levels of SRF, an associate of MYO that is known to enhance VSMC proliferation [7]. Despite consulting several databases, we did not find any binding sites for miR-223 on SMαA, MYO or SRF mRNAs; this argues in favour of an indirect effect of miR-223 on expression of these species. We also found that NFIA, an experimentally validated miR-223 specific target known to be expressed in VSMCs[33] was significantly downregulated at both messenger and protein levels in the presence of high Pi concentrations. Surprisingly, up- or downregulating miR-223 in VSMCs did not alter NFIA expression (data not shown). This strongly argues against a direct regulation of NFIA mRNA by miR-223 in our model. On the other hand, we show in our study that miR-223 upregulation affects the expression of two other recently described miR-223 targets, Mef2C [19]–[20] and RhoB [21]. Mef2c regulates the expression of MYO and affects VSMC migration and differentiation [18]. RhoB is a member of the Rho guanosine triphosphatases family of proteins which has been shown to increase VSMC contractivity and plays a part in the adaptation to hypoxia [22]. Our results thus suggest that miR-223 has a role in the VSMCs' complex response to Pi. Accelerated migration, a less structured and decreased actin cytoskeleton in the presence of high Pi, and a modulation of target genes Mef2c and RhoB could be linked (at least in part) to upregulation of miR-223. In contrast, high Pi treatment was associated with low VSMC proliferation. The resulting increase in miR-223 could be a compensatory mechanism for alleviating (at least in part) the cellular damage.

In conclusion, our data demonstrate that elevated Pi has strong impact on VSMC calcification, proliferation and migration. These effects are concomitant with deregulation of miRNAs and marker genes. In our *in vitro* and *in vivo* models, we confirmed the previously described impacts of miR-143 and miR-145 on normal and pathological cardiovascular events. We also highlighted (for the first time) a potential role for miR-223 in these conditions. Our results suggest that miR-143, miR-145 and miR-223 are potential biomarkers of vascular calcification. To date, only one miRNA (miR-125b[34]) has been described as a marker for these disorders. This observation emphasizes the importance of identifying other miRNAs with diagnostic value and better understanding their underlying molecular mechanisms of action.

## Materials and Methods

### Vascular smooth muscle cell culture

Human VSMCs were obtained by a modification of the explant method[35], detailed in Methods S1.

### Animals: diet, histological procedures

All experiments were performed on female mice (Charles Rivers, France). The animals were housed in temperature- and humidity-controlled polycarbonate cages with a 12-hour/12-hour light/dark cycle and were given standard chow diet (Teklad Global Diet 2016, Harlan, UK) and tap water *ad libitum*. The study was performed in two different mouse models: the C57 black wild-type (WT) mouse and the ApoE-KO mouse. The aorta was dissected down to the renal arteries and removed. Aortas were then frozen into liquid nitrogen and stored at −80°C until further use.

### Ethics Statement

All of the animal studies were conformed to the principles of the Directive 2010/63/EU of the European Parliament and all protocols were approved by our Institution's Animal Care and Use Committee (Comité Régional d'Ethique en Matière d'Expérimentation Animale de Picardie, CREMEAP) under PROTOCOL N° 2006/B7. Mice were anesthetized with ketamine and xylazine (100 mg/kg and 20 mg/kg, respectively), and all efforts were made to minimize suffering.

Human aortas were obtained after bypass surgery from patients afflicted with cardiovascular disorders (Pôle CTV of CHU Amiens, France) who gave informed written consent in accordance with French legislation, under PROTOCOL N°2009-19, CPP Nord-Ouest II.

### The VSMC mineralization assay

Mineralization of a VSMC monolayer (15,000 cells/well in 24-well plates) was induced using 1% FBS-containing medium (DMEM) supplemented with 3.5 mM Pi (control Pi concentration was 1.1 mM). Mineralization was detected and quantified according to an adaptation of the protocol described by Stanford et al[36]. Briefly, after fixation (in 50% ethanol for 5 min and 100% ethanol for 5 min), the VSMC monolayer was stained for 5 min with 40 mM Alizarin Red S (pH 4.2) at room temperature. The wells were rinsed thoroughly 3–4 times with water to remove unbound stain and then photographed. The deposited Alizarin-Ca^2+^ complexes were extracted by the addition of 10% cetylpiridinium chloride in 10 mM sodium phosphate (pH 7) at room temperature. Part of the extract was transferred to a 96-well plate and read at 570 nm. The monolayer's calcium content was normalized against the total protein content.

### Cell proliferation/viability assays

Vascular smooth muscle cells (2×10^4^ per well) were seeded in 96-well plates in DMEM. Cells were transfected for 48 h with pre-miR-223/anti-miR-223 or treated with Pi for 10 days. The VSMCs' metabolic activity was quantified using WST-1 reagent (Roche). Ten microliters of WST-1 were added to each well. After a 2-hour incubation, the absorbance at 415 nm (reference wavelength: 550 nm) was measured in an EnVision multilabel reader (PerkinElmer). The absorbance was converted into a cell number by reference to known numbers of cultured cells. The bromodeoxyuridine (BrdU) ELISA assay (Roche Applied Science) was used to quantify cell proliferation, according to manufacturer's instructions. Briefly, BrdU reagent was added to the medium for the final 24 h of the experiment. The medium was removed and the cell monolayer was washed and processed for colorimetric detection by measuring optical absorbance at 450 nm (Perkin Elmer).

### Immunofluorescence staining and protein quantitation

Fixed cells were permeabilized for 10 min at room temperature with 0.1% Triton X-100 in PBS containing 1% BSA and incubated with 5% BSA in PBS for 1 h at RT. After washing with PBS, VSMC monolayers were incubated overnight at 4^°^C with specific primary antibodies against SMαA (Abcam), SRF (Abcam), MYO (Santa Cruz), KLF4 (Abcam), Mef2C (Santa Cruz) and RhoB (Santa Cruz). After extensive washing, the cells were incubated with secondary antibody (anti-rabbit IgG or anti-mouse IGg conjugated to AlexaFluor 488, Sigma-Aldrich) for another 1 h. After washing in PBS, coverslips were mounted with Vectashield (Vector Laboratories). Laser-induced fluorescence was observed with a confocal microscope (FV1000, Olympus). Images were obtained with an Axioplan2 microscope (Carl Zeiss). Briefly, Z-stack images were acquired on a LSM 510 confocal microscope (Carl Zeiss). Dye intensity was quantified on a maximal projection of Z-stack images after thresholding, using the Zeiss LSM and ImageJ software. These data are presented as the antibodies' mean fluorescence intensity.

### Protein preparation and western blotting

After 10 days of treatment with 3.5 mM of Pi, Western blotting was performed as detailed in Methods S1. Protein loading was normalized against the β-actin signal. Band intensity was quantified versus a control sample, using ImageJ software.

### Migration assay (Boyden chamber)

The migration of VSMCs was assessed after adequate treatments in a Boyden chamber assay (NeuroProbe, Inc). Briefly, VSMCs were grown to ∼70% confluence and rendered quiescent in DMEM containing 0.2% BSA for 12 h. On the same day, polycarbonate membranes with 8.0 µm pores (Nucleopore Corp) were coated with Matrigel (Sigma), as described previously [37]. The next day, cultured cells were removed from plates using cell dissociation solution (Sigma) and suspended in DMEM containing 0.2% BSA. Next, 215 µL of DMEM containing 0.2% BSA and 10 ng/mL of PDGF-BB (R&D systems) were added to the lower chamber. After placement of the Matrigel-coated membrane between the chambers, 500 µL of media with suspended VSMCs were loaded in the upper chamber (50000 cells/chamber). After a 5 h incubation, cells were fixed with 100% ice-cold methanol for 2 min and stained with Giemsa (Fluka) for 2 min. Non-invading cells were gently removed from the upper part of membrane. The remaining cells were visualized using a Leica DM2500 microscope. Stained, migrated cells were counted using the Histolab® software (Histolab).

### Monolayer wound healing assay

Cells were grown in 10% FBS medium on 60 mm plates. After sub-confluence, the cells were wounded by scraping the monolayer with a yellow P200 pipette tip and grown with appropriate treatment for 48 h. The extent of healing was observed at 48 h using an inverted microscope in phase contrast mode (40×, Leica). Three to four different locations were visualized and photographed. Cells having migrated across the white lines were counted using Histolab® software.

### RNA isolation and real-time PCR

RNAs from VSMCs and mouse aorta were isolated with the *mir*Vana™ Isolation Kit (Applied Biosystems) as per the manufacturer's instructions. For miRNAs, Applied Biosystems Taqman assays were used. For phenotypic marker genes, 1 µg of DNAse-I-digested RNA was reverse-transcribed using a High-Capacity cDNA Synthesis Kit (Applied Biosystems). Quantitative real-time PCRs were then run on a StepOnePlus system (Applied Biosystems) using the primers described in Table S1. The U6 small nuclear RNA and GAPDH mRNA were used as endogenous control for miRNA and mRNAs, respectively.

### Transfection of pre-miR-143/223 or anti-miR-143/223

The siPORT NeoFX transfection agent (Applied Biosystems) was used for miRNA knock-down and overexpression, according to manufacturer's instructions. First, VSMCs were seeded in 6-well culture plates (Corning) and transfected with irrelevant scramble, anti-miR-223/143 or pre-miR-223/143 ((Applied Biosystems). Cells were collected and processed for further analysis after 48 h. Figure S7 shows that VSMCs transfected with pre-mir-223 or anti-mir-223 significantly up-regulated or reduced the expression of miR-223, respectively. Accordingly, the expression of miR-143 was unaffected in either of the conditions.

### Statistical analysis

Data are shown as the mean ± standard deviation (SD). Statistical significance was determined by two-tailed student's *t*-test (except for mice data which were examined by a one-way ANOVA). The statistical significance of the test results are denoted as follows: *p*<0.05 (*), *p*<0.01 (**) and *p*<0.005 (***).

## Supporting Information

Methods S1 (DOCX)

Table S1 (DOCX)

Figure S1 High Pi treatment does not induce cell apoptosis in VSMC.
(DOCX)

Figure S2 miR-223 is expressed in VSMCs, and its expression is increased by high Pi.
(DOCX)

Figure S3 Expression of miR-223 relative to miR-143 in control or Pi treated VSMCs.
(DOCX)

Figure S4 Pi treatment reduced actin cytoskeleton in VSMCs.
(DOCX)

Figure S5 Microscopic images showing VSMC SMα-actin and cortactin staining after high Pi treatment.
(DOCX)

Figure S6 Effect of a combination of over-expression of miR-223 and high Pi on VSMC migration.
(DOCX)

Figure S7 Effect of pre-miR-223 and anti-miR-223 on expression of miR-143 and miR-223 in VSMCs.
(DOCX)
