# Supplementary material for: Inorganic Phosphate Accelerates the Migration of Vascular Smooth Muscle Cells: Evidence for the Involvement of miR-223
Source: PLoS One. 2012 Oct 18;7(10):e47807. doi: 10.1371/journal.pone.0047807 (PMC3475714; doi:10.1371/journal.pone.0047807)
Supplement: Methods S1 — (DOCX) [file pone.0047807.s001.docx]

**Inorganic phosphate accelerates the migration of vascular smooth muscle cells: evidence for the involvement of miR-223.**

Ashraf Yusuf Rangrez**^1,2 ,$^**, Eléonore M’Baya-Moutoula**^1,2 ,$^**, Valérie Metzinger-Le Meuth**^1,4, #^**, Lucie Hénaut**^1,2, #^**, Mohamed Seif el Islam Djelouat**^1,2^**, Joyce Benchitrit**^1,2^**, Ziad A. Massy**^1,2,3^**, Laurent Metzinger**^1,2,*^**

**Online Supplemental Data**

**Supplementary Methods:**

*VSMC culture*

Briefly, segments of aorta were collected from human patients and stored in Dulbecco’s modified Eagle’s medium (DMEM, Sigma). Endothelium was removed and medial tissue was separated. Small pieces of tissue (1mm^2^) were evenly distributed in Petri dishes and cultured for 2-3 weeks in DMEM supplemented with 15% foetal bovine serum (FBS; Gibco). Cells that migrated from the explants were collected by trypsinization and maintained in DMEM containing 15% FBS supplemented with 1x penicillin G/streptomycin (Sigma) and 1x glutamax (Gibco). The media were replaced every two to three days. Only cells between passages 3 to 10 were used for the experiments.

*Apoptosis measurement*

Cells were cultured in a 4-well culture dish (25 × 10^4^ cells/well) and treated with 3.5 mM Pi for 10 days. Then, cells were trypsinized and suspended into solution containing Annexin V-FITC and Propidium Iodide (Roche, Switzerland), according to the manufacturer’s instructions. After incubation for 1 hr in the dark, cells were subjected to flow cytometry assay.

*In situ hybridization (ISH)*

ISH was carried out in VSMCs by mercury LNA microRNA ISH kit (FFPE, Exiqon) using manufacturer’s instructions. We used 5’-DIG labelled U6 control, scramble microRNA (negative control) and a double digoxigenin (DIG)-labelled mercury Locked Nucleic Acid (LNA) probe (LNA™ microRNA detection probe, Exiqon, Vedbaek, Denmark) specific for human miRNA-223. Cell culture was performed in an RNase-free environment.

*α-actin immunostaining*

Immunostaining was performed with paraformaldehyde-fixed VSMCs on small coverslips. Fixed cells were permeabilized for 10 min at room temperature with 0.1% Triton X-100 in PBS containing 1% BSA. Endogenous peroxydase activity was blocked by 0.3% H_2_O_2_ in PBS with 1% BSA for 10 min at room temperature followed by 30 min incubation with 1% BSA in PBS for 30 min at room temperature to block non-specific antibody binding. VSMC monolayers were then incubated for 1h at room temperature with primary antibody (mouse monoclonal anti-human-α-actin, Santa Cruz Biotechnology SC-32251, 1:100 dilution from original unit) in 1% BSA in PBS. VSMCs were then rinsed with 1% BSA in PBS and incubated with secondary antibody (anti-mouse IgG (whole molecule)-peroxydase produced in goat, Sigma A4416, 1:500 dilution from original unit) for 1h at room temperature. Coverslips were then thoroughly washed in PBS, and incubated with peroxydase substrate from the kit (Vector® *NovaRED^TM^* SK-4800) at room temperature until development of suitable coloration as per manufacturer’s instructions. Cells were counterstained with hematoxylin solution and mounted on glass slides using VectaMount^TM^ mounting medium (Vector® H-5000). Images were taken at 40X magnification using a Leica DM 2500 microscope.

*Co-immunofluorescence*

Co-immunofluorescence was performed using cortactin (CTTN) and α-actin antibodies to determine podosome formation and actin cytoskeleton respectively. Fixed cells were permeabilized for 10 min at room temperature with 0.1% Triton X-100 in PBS containing 1% BSA and incubated with 5% BSA in PBS for 1h at room temperature to block non-specific antibody binding. Following washing with PBS, VSMC monolayers were then incubated for overnight with 1:250 diluted primary antibody (rabbit monoclonal, anti-human-cortactin, Abcam) at 4 ^o^C. After three washes with PBS, cells were incubated with primary α-actin antibody as mentioned above. Antibodies conjugated to Alexa Fluor 488 and 568 were used as secondary antibodies and coverslips were mounted with Mounting Medium for Fluorescence (Vectashield; Vector Laboratories). Images were obtained with an Axioplan2 microscope (Carl Zeiss, Inc.) using Axiovision software (Carl Zeiss, Inc.) at room temperature.

*Western blot analysis*

For total proteins preparation, cells were harvested by trypsinization and centrifuged at 600 × *g* for 10 min at 4 °C. The cell pellets were resuspended in lysis buffer supplemented with protease inhibitor mixture (1 X, Roche Diagnostics). Homogenate was centrifuged at 1500 × *g* at 4 °C to remove cells debris and nuclei. Each protein sample (50 µg) was resolved by sodium dodecyl sulphate–polyacrylamide gel electrophoresis, transferred onto a polyvinylidene difluoride membrane, and incubated with a rabbit polyclonal antibody against NFI-A (ab41851, 1 : 500, Abcam ), or β-actin (1 : 2000; Sigma-Aldrich). The signals were detected by incubation with labelled secondary antibodies using the ECL Detection System (GE Healthcare, Little Chalfont, UK).
