# Supplementary material for: Inorganic Phosphate Accelerates the Migration of Vascular Smooth Muscle Cells: Evidence for the Involvement of miR-223
Source: PLoS One. 2012 Oct 18;7(10):e47807. doi: 10.1371/journal.pone.0047807 (PMC3475714; doi:10.1371/journal.pone.0047807)
Supplement: Table S1 — (DOCX) [file pone.0047807.s002.docx]

**smooth muscle cells: evidence for the involvement of miR-223.**

Ashraf Yusuf Rangrez**^1,2 ,$^**, Eléonore M’Baya-Moutoula**^1,2 ,$^**, Valérie Metzinger-Le Meuth**^1,4, #^**, Lucie Hénaut**^1,2, #^**, Mohamed Seif el Islam Djelouat**^1,2^**, Joyce Benchitrit**^1,2^**, Ziad A. Massy**^1,2,3^**, Laurent Metzinger**^1,2,*^**

**Supplementary Table:**

**Supplementary Table 1 :** Primer details for the phenotypic marker genes used in current study for real-time PCR analysis of respective genes.

| **Gene name** | **Primer name** | **Sequence (5'-3')** | **Tm (C)** | **Product length** | **NCBI reference** |
| --- | --- | --- | --- | --- | --- |
| Myocardin | MYO_fw | CCCAGCACCCCCATAGC | 58 | 64 | NM_001146312 |
|  | MYO_rv | GGTTCTTACTGTCACCCAAGGATT | 58 |  |  |
| Serum response factor | SRF_fw | CGACCTTCAGCAAGAGGAAGAC | 59 | 165 | NM_003131 |
|  | SRF_rv | AGTGCCTTGCCGGTCTCA | 59 |  |  |
| Kruppel-like factor 4 | KLF4_fw | CCACACAGGTGAGAAACCTTACC | 59 | 104 | NM_004235 |
|  | KLF4_rv | CGGTGCCCCGTGTGTTTA | 60 |  |  |
| Kruppel -like factor 5 | KLF5_fw | TTCTTCCACAACAGGCCACTT | 58 | 61 | NM_001730 |
|  | KLF5_rv | GGACTTCCAGGCTCTGAGCTT | 59 |  |  |
| Cortactin | CTTN_fw | CAAGAGCATCAGACCCTTAAGGA | 59 | 66 | NM_005231 |
|  | CTTN_rv | TCCATAGCCATGGGAAGCTT | 58 |  |  |
| Nuclear factor 1A | NFIa_fw | CAGCATCACCGACCTGTCAT | 58 | 99 | NM_001134673 |
|  | NFIa_rv | GCCCAGGCTGCTGGATAA | 58 |  |  |
| Versican | VSCN_fw | TGGAAGGCACGGCAATCTA | 59 | 113 | NM_004385 |
|  | VSCN_rv | TGGCACACAGGTGCATACGT | 60 |  |  |
| MEF2c | MEF_fw | CTGGTGTAACACATCGACCTC | 59 | 77 | NM_002397.4 |
|  | MEF_rv | GATTGCCATACCCGTTCCCT | 60 |  |  |
| RhoB | RHB_fw | GAGAACATCCCCGAGAAG-G | 58 | 88 | NM_004040.2 |
|  | RHB_rv | CTTCCTTGGTCTTGGCAGAG | 60 |  |  |
| Platelet-derived growth factor receptor-alpha | PDGFRa_fw | CTTATTGTCCTGGTTGTCATTTGG | 58 | 64 | NM_006206 |
|  | PDGFRa_rv | CAATGACCCTCCAGCGAATT | 59 |  |  |
| Glycerinaldehyd-3-phosphate-Dehydrogenase | GAPDH_fw | ATGGAAATCCCATCACCATCTT | 58 | 57 | NM_002046 |
|  | GAPDH_rv | CGCCCCACTTGATTTTGG | 58 |  |  |
| Smooth muscle alpha actin | SMaA_fw | TGCCTGATGGGCAAGTGA | 58 | 51 | NM_001613 |
|  | SMaA_rv | CTGGGCAGCGGAAACG | 58 |  |  |
