# Supplementary material for: Inorganic Phosphate Accelerates the Migration of Vascular Smooth Muscle Cells: Evidence for the Involvement of miR-223
Source: PLoS One. 2012 Oct 18;7(10):e47807. doi: 10.1371/journal.pone.0047807 (PMC3475714; doi:10.1371/journal.pone.0047807)
Supplement: Figure S1 — High Pi treatment does not induce cell apoptosis in VSMC. (DOCX) [file pone.0047807.s003.docx]

**smooth muscle cells: evidence for the involvement of miR-223.**

Ashraf Yusuf Rangrez**^1,2 ,$^**, Eléonore M’Baya-Moutoula**^1,2 ,$^**, Valérie Metzinger-Le Meuth**^1,4, #^**, Lucie Hénaut**^1,2, #^**, Mohamed Seif el Islam Djelouat**^1,2^**, Joyce Benchitrit**^1,2^**, Ziad A. Massy**^1,2,3^**, Laurent Metzinger**^1,2,*^**

**Online Supplemental Data**


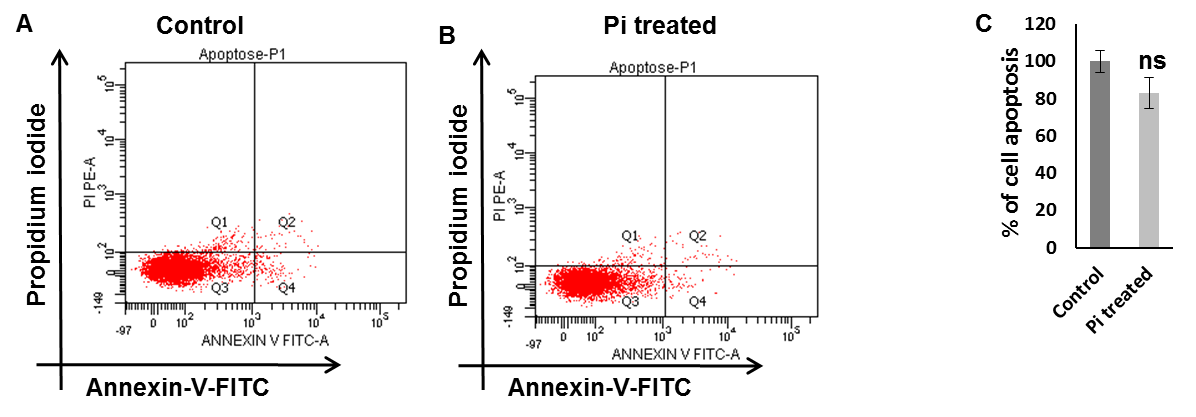


**Supplemental Figure S1. High Pi treatment does not induce cell apoptosis in VSMC.** Cell apoptosis was detected by flow cytometry with Annexin-FITC/PI double staining. A) Untreated cell control. B) Cells treated with 3.5 mM of Pi for 10 days. C) Percentage of apoptotic cells. Data represent the mean of three independent experiments. Statistical significance was determined by two tailed student’s *t*-test.
