# Supplementary material for: Inorganic Phosphate Accelerates the Migration of Vascular Smooth Muscle Cells: Evidence for the Involvement of miR-223
Source: PLoS One. 2012 Oct 18;7(10):e47807. doi: 10.1371/journal.pone.0047807 (PMC3475714; doi:10.1371/journal.pone.0047807)
Supplement: Figure S2 — miR-223 is expressed in VSMCs, and its expression is increased by high Pi. (DOCX) [file pone.0047807.s004.docx]

Ashraf Yusuf Rangrez**^1,2 ,$^**, Eléonore M’Baya-Moutoula**^1,2 ,$^**, Valérie Metzinger-Le Meuth**^1,4, #^**, Lucie Hénaut**^1,2, #^**, Mohamed Seif el Islam Djelouat**^1,2^**, Joyce Benchitrit**^1,2^**, Ziad A. Massy**^1,2,3^**, Laurent Metzinger**^1,2,*^**

**Online Supplemental Data**


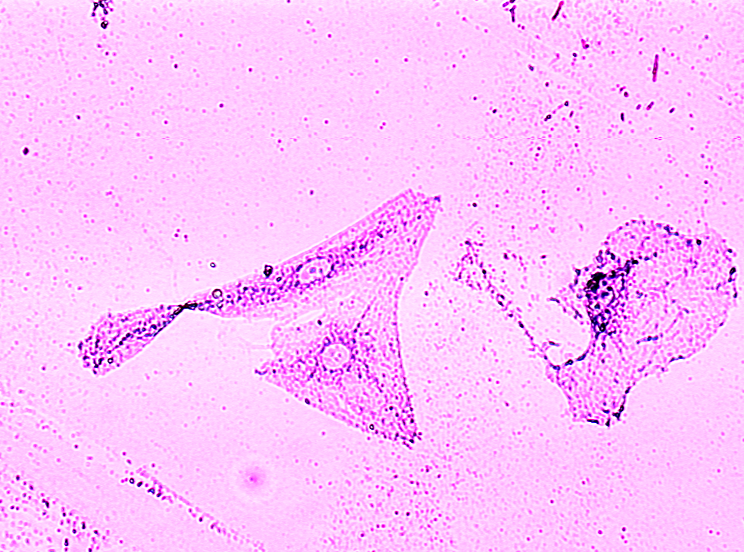

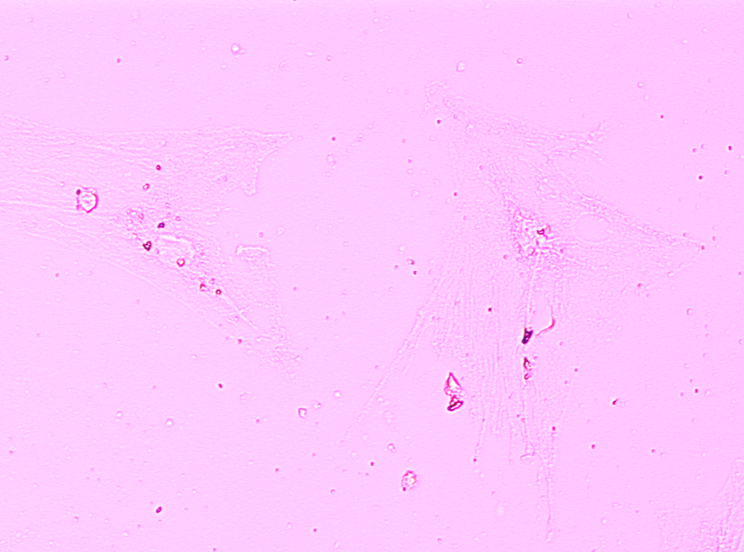

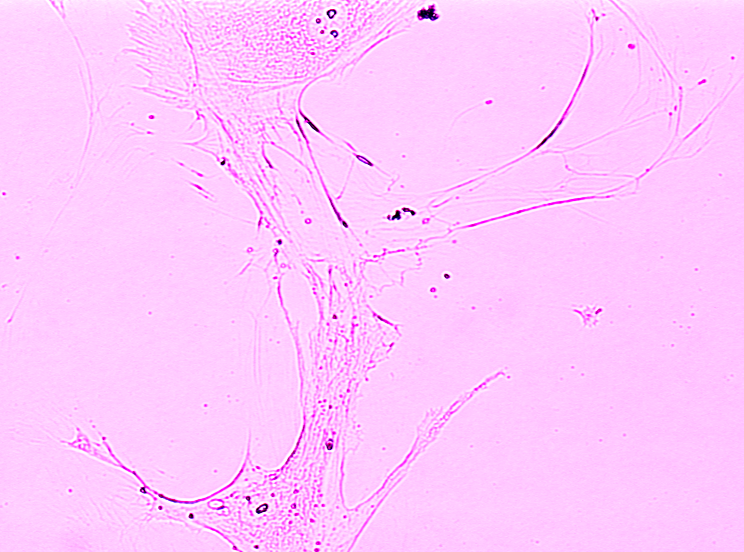


**Control**

**Pi-treated**

**Scramble**

**Supplemental Figure S2: miR-223 is expressed in VSMCs, and its expression is increased by high Pi.** miR-223 expression is validated by ISH in VSMCs. Treatment by 3,5 mM Pi for 10 days markedly increased miR-223 staining when compared to control incubated with 1,1 mM Pi. Scramble probe is used as negative control. Magnification 40 X. One representative experiment shown out of three independent experiments.
