# Supplementary material for: Inorganic Phosphate Accelerates the Migration of Vascular Smooth Muscle Cells: Evidence for the Involvement of miR-223
Source: PLoS One. 2012 Oct 18;7(10):e47807. doi: 10.1371/journal.pone.0047807 (PMC3475714; doi:10.1371/journal.pone.0047807)
Supplement: Figure S3 — Expression of miR-223 relative to miR-143 in control or Pi treated VSMCs. (DOCX) [file pone.0047807.s005.docx]

**smooth muscle cells: evidence for the involvement of miR-223.**

**smooth muscle cells: evidence for the involvement of miR-223.**

Ashraf Yusuf Rangrez**^1,2 ,$^**, Eléonore M’Baya-Moutoula**^1,2 ,$^**, Valérie Metzinger-Le Meuth**^1,4, #^**, Lucie Hénaut**^1,2, #^**, Mohamed Seif el Islam Djelouat**^1,2^**, Joyce Benchitrit**^1,2^**, Ziad A. Massy**^1,2,3^**, Laurent Metzinger**^1,2,*^**

**Online Supplemental Data**


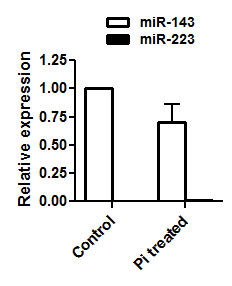

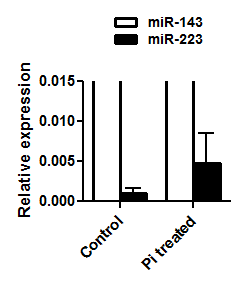

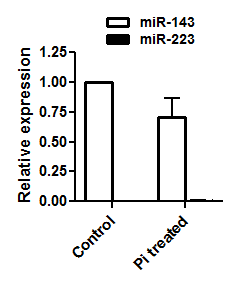

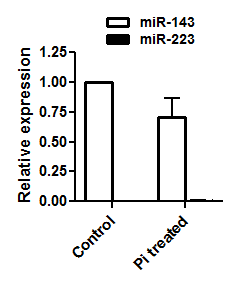


*

p = 0.16

**Supplemental Figure S3: Expression of miR-223 relative to miR-143 in control or Pi treated VSMCs.** Expression of miR-143 and miR-223 was determined by quantitative real-time PCR, and was normalized to the expression of miR-143 in control cells. Data represented is mean of three independent experiments. Statistical significance was determined by two tailed student’s *t*-test (*n* = 3 ± SD, **P* < 0.05)
