# Supplementary material for: Inorganic Phosphate Accelerates the Migration of Vascular Smooth Muscle Cells: Evidence for the Involvement of miR-223
Source: PLoS One. 2012 Oct 18;7(10):e47807. doi: 10.1371/journal.pone.0047807 (PMC3475714; doi:10.1371/journal.pone.0047807)
Supplement: Figure S4 — Pi treatment reduced actin cytoskeleton in VSMCs. (DOCX) [file pone.0047807.s006.docx]

**smooth muscle cells: evidence for the involvement of miR-223.**

Ashraf Yusuf Rangrez**^1,2 ,$^**, Eléonore M’Baya-Moutoula**^1,2 ,$^**, Valérie Metzinger-Le Meuth**^1,4, #^**, Lucie Hénaut**^1,2, #^**, Mohamed Seif el Islam Djelouat**^1,2^**, Joyce Benchitrit**^1,2^**, Ziad A. Massy**^1,2,3^**, Laurent Metzinger**^1,2,*^**


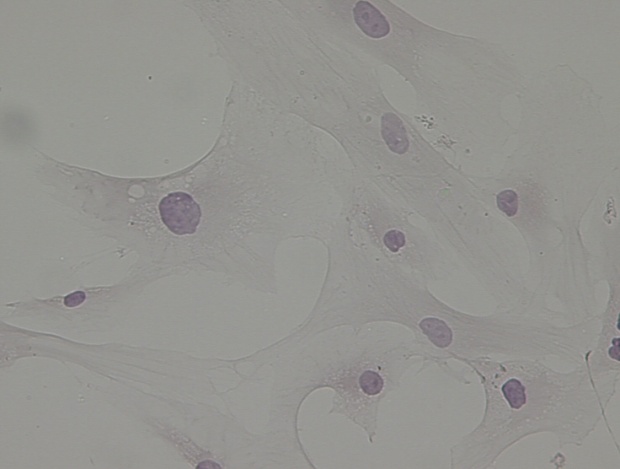

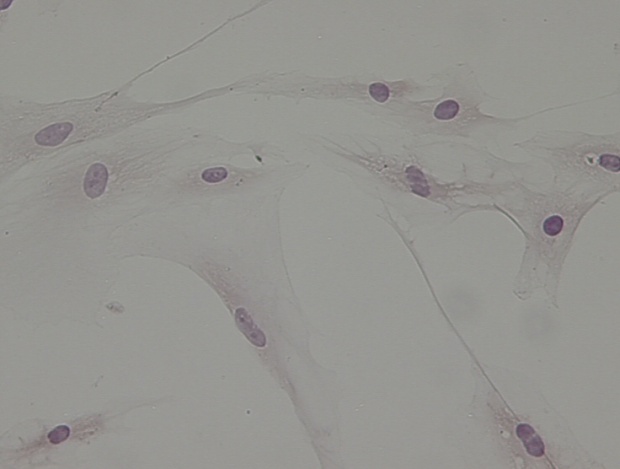

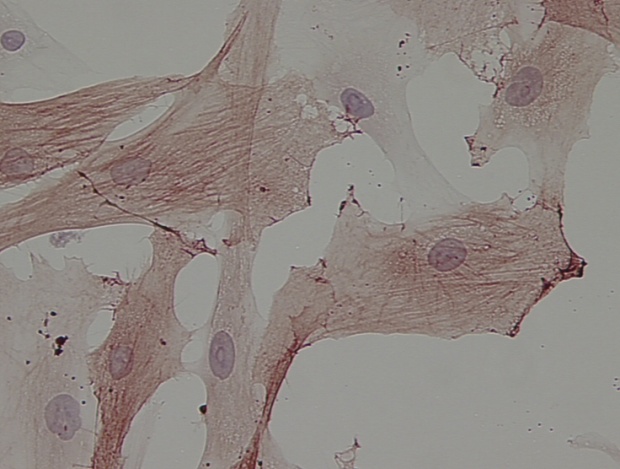

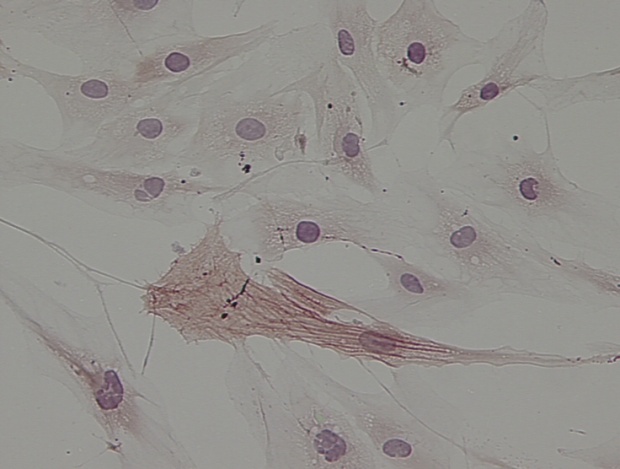


**I**

**II**

**III**

**IV**

**Supplemental Figure S4: Pi treatment reduced actin cytoskeleton in VSMCs.** Control (I and II, 1,1 mM Pi) and 3.5 mM Pi (III and IV) VSMCs were immunostained after 10 days treatment using smooth-muscle α-actin antibody. Images I and III represents negative controls from respective groups where no antibody was added during staining. On the other hand, images II and IV clearly demonstrate that 3,5 mM Pi treatment drastically reduced actin cytoskeleton and affected morphology of VSMCs. One representative experiment shown out of two independent experiments.
