# Supplementary material for: Inorganic Phosphate Accelerates the Migration of Vascular Smooth Muscle Cells: Evidence for the Involvement of miR-223
Source: PLoS One. 2012 Oct 18;7(10):e47807. doi: 10.1371/journal.pone.0047807 (PMC3475714; doi:10.1371/journal.pone.0047807)
Supplement: Figure S5 — Microscopic images showing VSMC SMα-actin and cortactin staining after high Pi treatment. (DOCX) [file pone.0047807.s007.docx]

**smooth muscle cells: evidence for the involvement of miR-223.**

Ashraf Yusuf Rangrez**^1,2 ,$^**, Eléonore M’Baya-Moutoula**^1,2 ,$^**, Valérie Metzinger-Le Meuth**^1,4, #^**, Lucie Hénaut**^1,2, #^**, Mohamed Seif el Islam Djelouat**^1,2^**, Joyce Benchitrit**^1,2^**, Ziad A. Massy**^1,2,3^**, Laurent Metzinger**^1,2,*^**

**
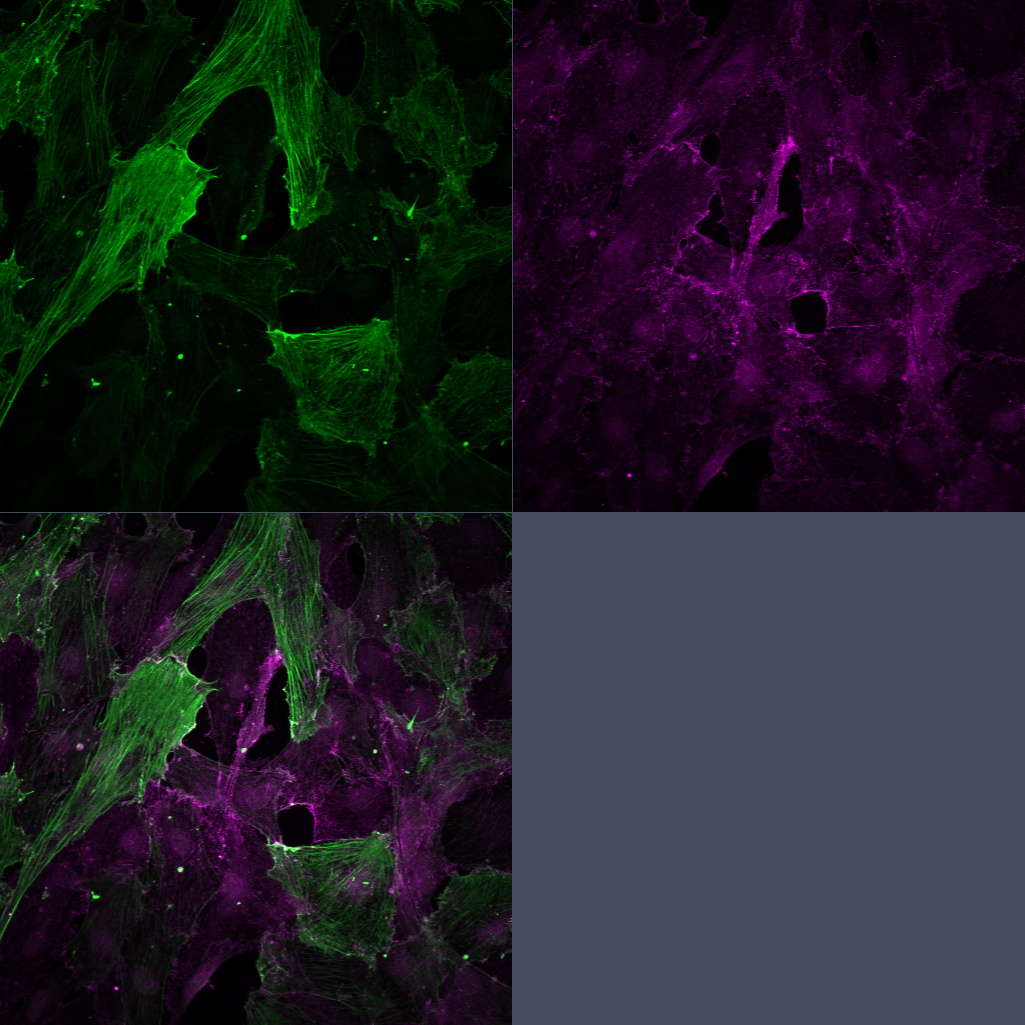

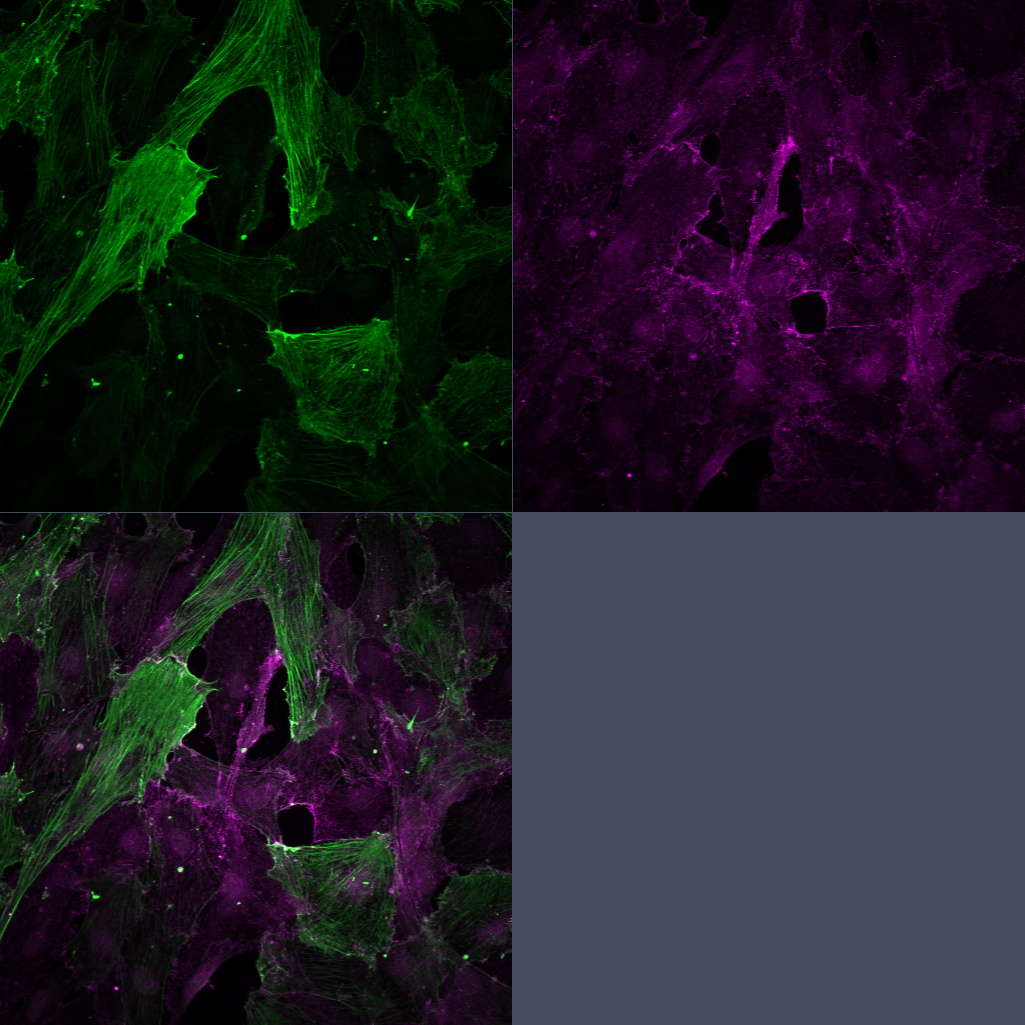

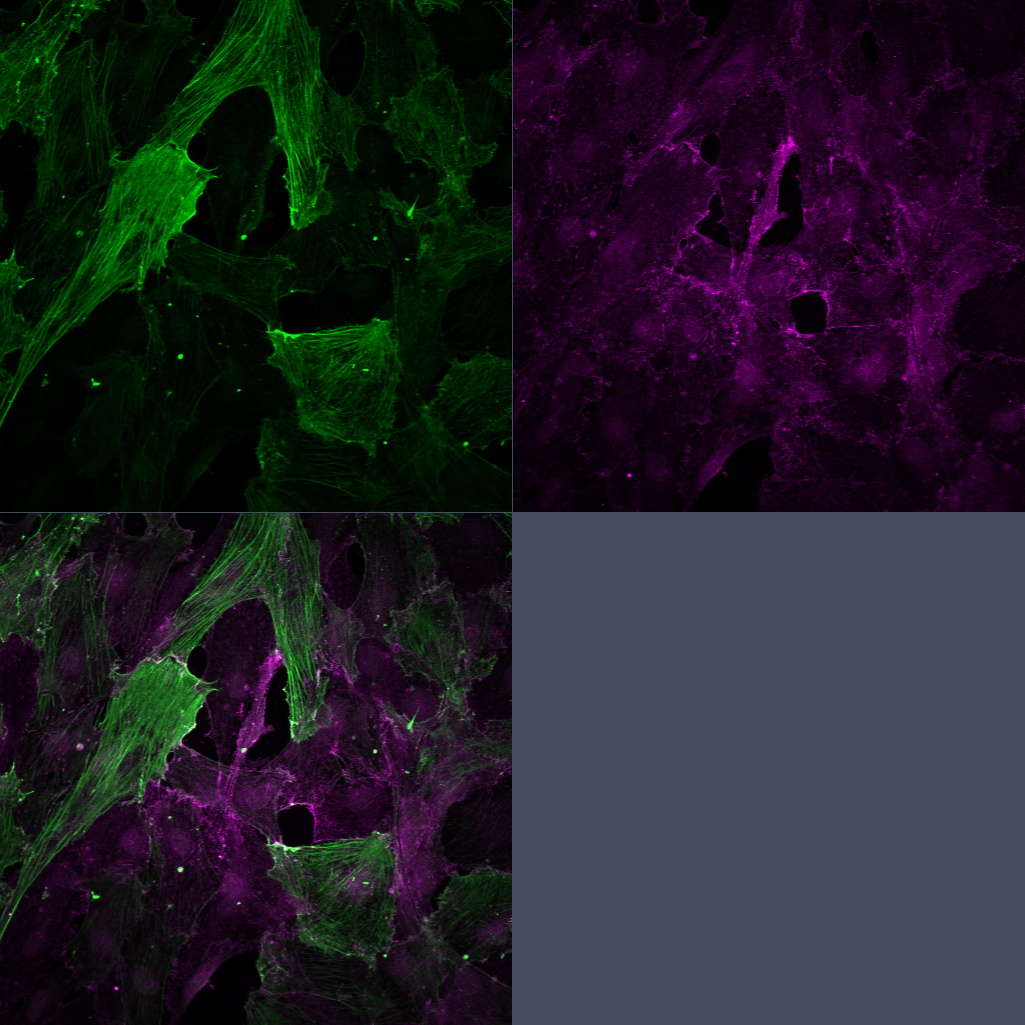

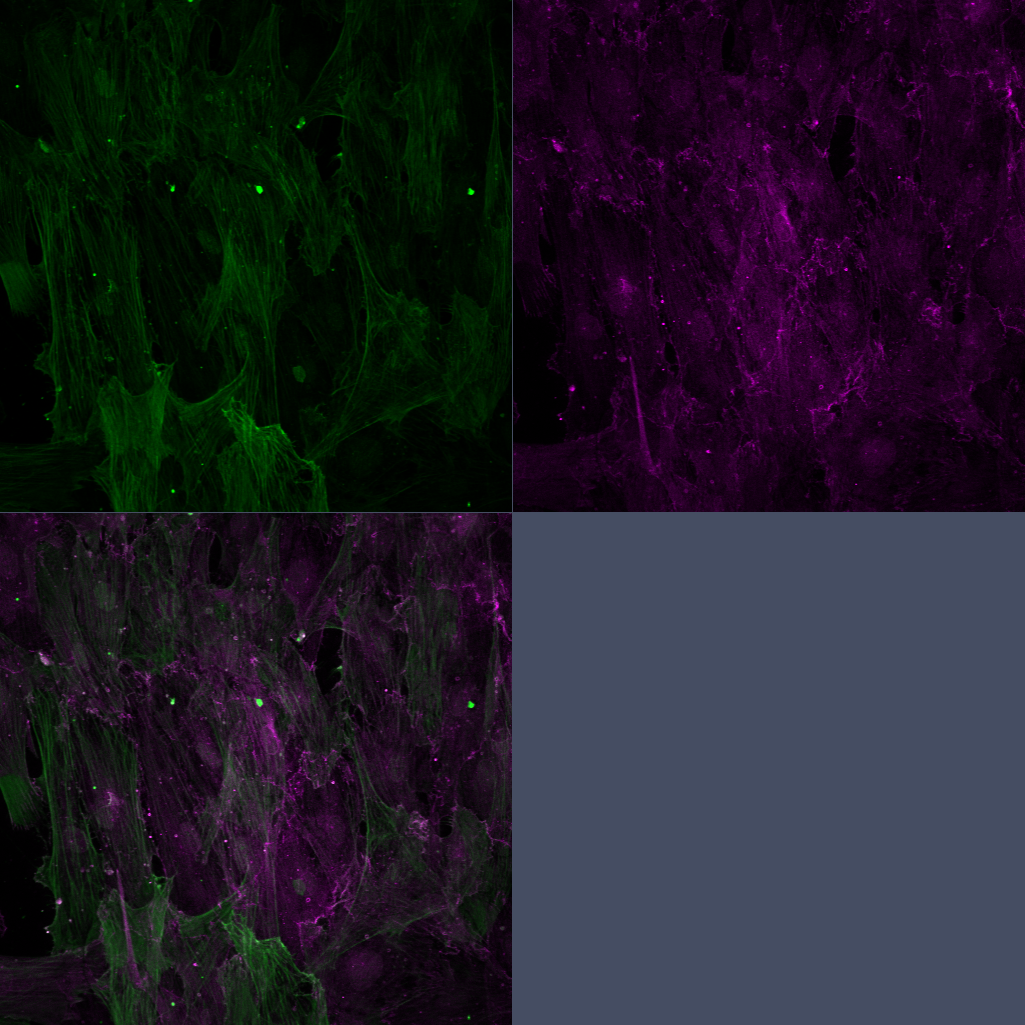

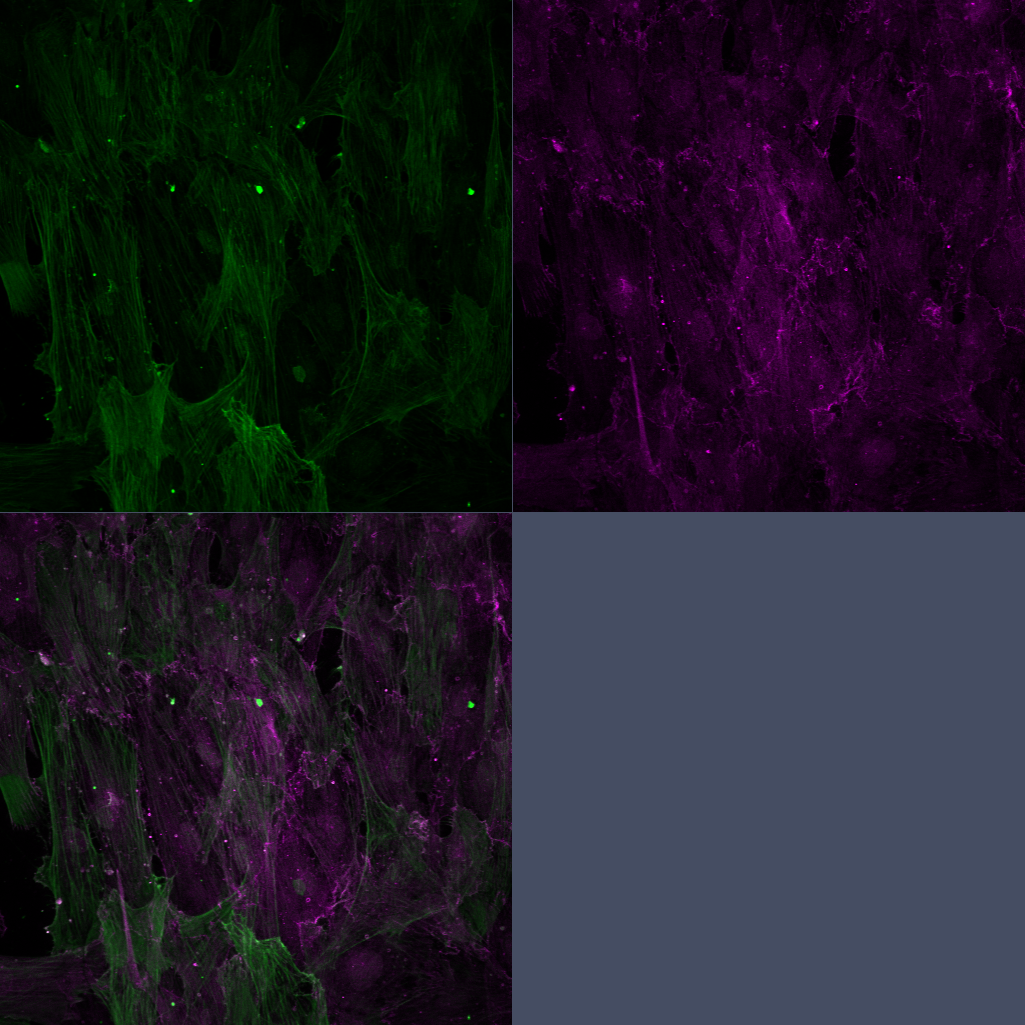
**

**Cortactin**

**SM α-actin**

**Merge**

**Control**

**
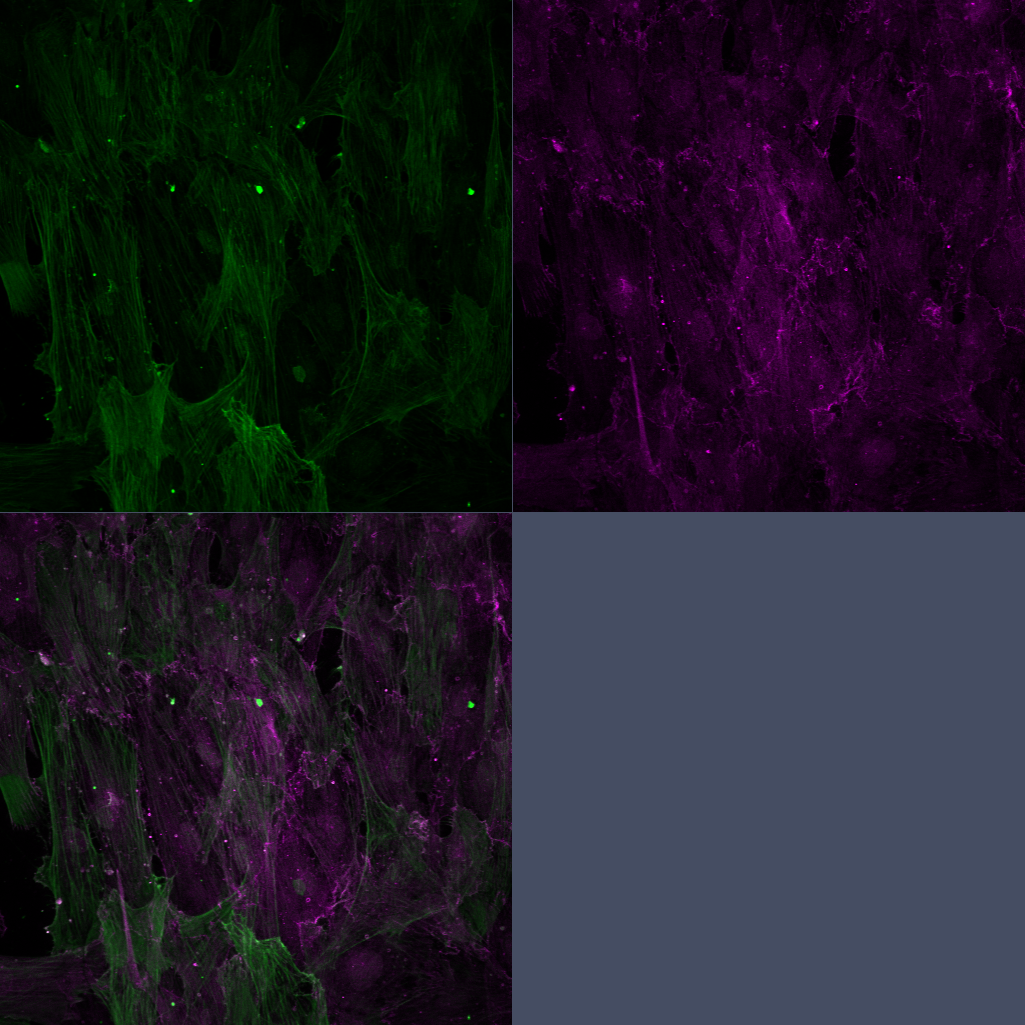
**

**Pi treated**

**Supplemental figure S5. Microscopic images showing VSMC α-actin (SM α-actin) and cortactin staining after high Pi treatment.** Cortactin and SMα-actin staining of VSMCs after 10 days of Pi treatment to visualise the podosomes (indicated by small arrowheads). This figure demonstrates that the cortactin stained, podosome rich VSMCs are deprived of SM α-actin further confirming the results shown in figure 2B. Representative figure from two independent experiments.
