# Supplementary material for: Inorganic Phosphate Accelerates the Migration of Vascular Smooth Muscle Cells: Evidence for the Involvement of miR-223
Source: PLoS One. 2012 Oct 18;7(10):e47807. doi: 10.1371/journal.pone.0047807 (PMC3475714; doi:10.1371/journal.pone.0047807)
Supplement: Figure S6 — Effect of a combination of over-expression of miR-223 and high Pi on VSMC migration. (DOCX) [file pone.0047807.s008.docx]

**smooth muscle cells: evidence for the involvement of miR-223.**

Ashraf Yusuf Rangrez**^1,2 ,$^**, Eléonore M’Baya-Moutoula**^1,2 ,$^**, Valérie Metzinger-Le Meuth**^1,4, #^**, Lucie Hénaut**^1,2, #^**, Mohamed Seif el Islam Djelouat**^1,2^**, Joyce Benchitrit**^1,2^**, Ziad A. Massy**^1,2,3^**, Laurent Metzinger**^1,2,*^**

**Supplemental figure S6. Effect of a combination of over-expression of miR-223 and high Pi on VSMC migration.** To upregulate and knock-down the expression of miR-143, miR-145 and miR-223, VSMCs were transfected for 48 h with corresponding pre-miRs and anti-miRs, respectively. Pi treated cells were incubated with 3.5 mM Pi for 48 h. Pi + Anti-miR-223 cells were treated for 48 h by a combination of 3.5 mM Pi and anti-miR-223. Scrambled, unrelated miRNA was used as a control. The migration rate was studied by wound healing assay. Migration rate of VSMCs is represented by the % of migration rate, the scrambled control being put at 100. Data represent the mean of three independent experiments. Statistical significance was determined by two tailed student’s *t*-test (*n* = 3 ± SD, ***P* < 0.01; ****P* < 0.001).
