# Supplementary material for: Inorganic Phosphate Accelerates the Migration of Vascular Smooth Muscle Cells: Evidence for the Involvement of miR-223
Source: PLoS One. 2012 Oct 18;7(10):e47807. doi: 10.1371/journal.pone.0047807 (PMC3475714; doi:10.1371/journal.pone.0047807)
Supplement: Figure S7 — Effect of pre-miR-223 and anti-miR-223 on expression of miR-143 and miR-223 in VSMCs. (DOCX) [file pone.0047807.s009.docx]

**smooth muscle cells: evidence for the involvement of miR-223.**

Ashraf Yusuf Rangrez**^1,2 ,$^**, Eléonore M’Baya-Moutoula**^1,2 ,$^**, Valérie Metzinger-Le Meuth**^1,4, #^**, Lucie Hénaut**^1,2, #^**, Mohamed Seif el Islam Djelouat**^1,2^**, Joyce Benchitrit**^1,2^**, Ziad A. Massy**^1,2,3^**, Laurent Metzinger**^1,2,*^**


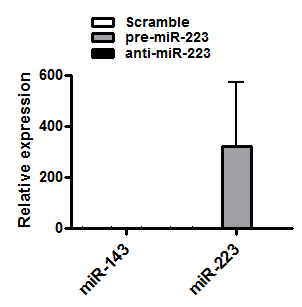

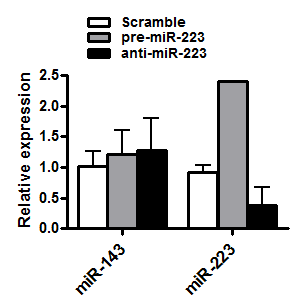

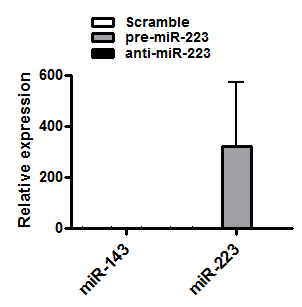

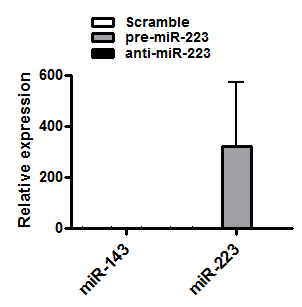


**

**

**Supplemental figure S7. : Effect of pre-miR-223 and anti-miR-223 on expression of miR-143 and miR-223 in VSMCs.** VSMCs were transfected using pre-miR-223 and anti-miR-223 to up-regulate and knock-down the expression of miR-223 respectively. After 48h of transfection, cells were collected and expression of miR-143 and miR-223 was determined by quantitative real-time PCR. Data represent the mean of four independent experiments. Statistical significance was determined by two tailed student’s *t*-test (*n* = 3 ± SD, ***P* < 0.01).
